# Endovascular thrombectomy versus medical management on outcomes with infarct volumes more than 70 mL

**DOI:** 10.1002/acn3.52124

**Published:** 2024-06-10

**Authors:** Nannan Han, Xiaobo Zhang, Yu Zhang, Yu Liu, Haojun Ma, Hanming Ge, Yanfei Wang, Shilin Li, Xudong Yan, Tengfei Li, Yulun Wu, Juan Ma, Wenzhen Shi, Gejuan Zhang, Ye Tian, Mingze Chang

**Affiliations:** ^1^ Department of Neurology The Affiliated Hospital of Northwest University Xi'an China; ^2^ The College of Life Sciences Northwest University Xi'an China; ^3^ School of Information Science and Technology Northwest University Xi'an China; ^4^ Xi'an Key Laboratory of Cardiovascular and Cerebrovascular Diseases The Affiliated Hospital of Northwest University Xi'an China; ^5^ Clinical Medical Research Center The Affiliated Hospital of Northwest University Xi'an China

## Abstract

**Objective:**

Endovascular thrombectomy (EVT) in patients with large infarct volume remains controversial. The aim of this study is to compare clinical outcomes between EVT and medical management in acute large vessel occlusion with infarct volumes larger than 70 mL on diffusion‐weighted magnetic resonance imaging (DWI).

**Methods:**

A prospective observational cohort study was conducted, including patients with anterior cerebral circulation occlusion due to ischemic stroke with infarct volumes larger than 70 mL within 24 h of onset between July 2018 and June 2023. Eligible patients were divided into two groups: the EVT group and the medical management (non‐EVT) group. The main outcomes were functional independence and mortality at 90 days. To assess clinical endpoints, we selected variables including age, NIHSS score, infarct volume, and occlusion location for 1:1 propensity score (PS) matching and PS adjustment using inverse probability of treatment weighting (IPTW).

**Results:**

Among the 131 identified patients (mean [SD] age, 69.9 [13.7] years; 58 female), the median infarct volume was 123.6 mL. Of these patients, 75 (57.3%) underwent EVT. After PS adjustment, EVT was not associated with functional independence (10.9% vs. 10.9%; *p* = 1.000) or mortality (43.5% vs. 47.8%; *p* = 0.675). Additionally, after PS adjustment using IPTW, EVT was also not associated with a functional independence (15.8% vs. 13.7%; *p* = 0.767) or mortality (46.8% vs. 44.0%; *p* = 0.762).

**Conclusion:**

This study provides real‐world evidence regarding infarct volumes larger than 70 mL, indicating that EVT does not provide benefits compared to medical management alone when considering age, NIHSS score, infarct volume, and occlusion location.

## Introduction

Large infarct core (LIC) accounts for approximately one‐fifth of the total incidence of large‐vessel occlusion stroke^1^ and had a high rate of mortality. Notably, several randomized clinical trials of thrombectomy, published in 2015, systematically excluded cases with LIC.^2^, ^3^, ^4^ Furthermore, patients with LIC were not considered for endovascular thrombectomy (EVT) within the extended time window of 16–24 h after stroke onset,^5^, ^6^ resulting in a missed opportunity for EVT in this specific cohort. It is noteworthy that several clinical guidelines do not endorse the use of EVT in patients who fall into this specific category.^7^, ^8^

Four recent randomized controlled trials have provided evidence of the effectiveness of EVT in patients with LIC compared with medical management.^9^, ^10^, ^11^, ^12^ All four studies used the computed tomography (CT) or diffusion‐weighted magnetic resonance imaging (DWI) by an Alberta Stroke Program Early Computed Tomographic Score (ASPECTS) as a semiquantitative criterion for patient inclusion. The studies showed a wide variation in the prognosis of patients undergoing EVT, with modified Rankin Scale scores (mRS) of 0–2 ranging from 14% to 30%, and mortality rates ranging from 18.0% to 38.4%, possibly due to differences in infarct volume. Patients with infarct volumes larger than 70 mL almost always had DWI‐ASPECTS of ≤5, but DWI‐ASPECTS ≤5 showed a wide range of infarct volumes in our previous study.^13^ A similar pattern was observed within the CT‐ASPECTS.^14^ Mehdi Bouslama found that in nearly 40% of cases, patients with low ASPECTS but limited baseline infarct volume achieve independence.^15^ Thus, a low ASPECTS score does not represent the totality of a large infarct volume (LIV).

We conducted this study to determine the efficacy of thrombectomy in patients with a LIV. We used DWI as a quantitative method to assess infarct volume, as opposed to the semiquantitative approach based on CT or DWI ASPECTS. Patients were selected based on a threshold of larger than 70 mL, aligning with the exclusion criteria utilized in the DEFUSE3 study.^5^ We hypothesized that EVT might demonstrate superior clinical efficacy when compared to medical management in patients with anterior circulation large vessel occlusion and an infarct volume larger than 70 mL.

## Methods

### Patients

The study population comprised all patients consecutively admitted to the neurology at the affiliated hospital of Northwest University, Xi'an No.3 hospital (China), due to anterior cerebral circulation ischemic stroke and receiving green channel for acute stroke treatment, between July 2018 and June 2023. The EVT group was preregistered on clinical trial (NCT03607565). All patients in the study underwent a standardized imaging protocol, which encompassed non‐enhanced CT and multimodal MRI, including DWI. The study participants were selected based on the following inclusion criteria: (1) age of 18 years or older, (2) presence of large vessel occlusion in the anterior circulation (including the internal carotid artery and M1‐M2 segment of the middle cerebral artery) confirmed by MRA, (3) onset‐to‐arrival time within 24 h. The time of stroke onset for wake‐up stroke was ascertained through calculation of the midpoint between the time of the last normal and the time when symptoms were detected.^16^ (3) infarct volume larger than 70 mL on DWI, and (4) pre‐thrombectomy DWI without brain edema or midline shift on FLAIR, while partial FLAIR high signals in the corresponding area of high signals on DWI are acceptable. The following exclusion criteria were also considered: (1) prior history of stroke with a mRS score of 3–5, (2) CT or MRI before thrombectomy demonstrating brain tissue swelling with midline shift in the EVT group, (3) known allergies (more severe than skin rash) to contrast agents, (4) evidence of acute intracranial hemorrhage in CT or MRI, (5) high risk of hemorrhage (platelet <40,000/μL), (6) DWI motion artifacts that make it difficult to accurately identify and measure the infarcted area, and (7) simultaneous occlusion in both anterior and posterior circulation.

The study received approval from the Institutional Review Board of the Affiliated Hospital of Northwest University (No. SYXSLL‐2018‐010), and informed consent or refusal for EVT was obtained from legally authorized representatives. The data supporting the findings of this study can be obtained from the corresponding author upon a reasonable request.

### Clinical data collection

Clinical, radiological, and treatment characteristics of patients were systematically collected in a prospective way. The EVT procedures utilized stent retrievers, aspiration devices, balloons, and/or stenting. All patients received medical management in accordance with local guidelines. Successful recanalization was defined as achieving a modified treatment in cerebral ischemia (mTICI) score of 2b or higher. The ultimate mTICI score was evaluated by a neuroradiologist with more than 10 years of experience, who was blinded to the clinical outcome results. CT scans were systematically performed 24 h after EVT, and these imaging data were subsequently evaluated by a neuroradiologist with 11 years of experience, ensuring blindness to both the procedure and clinical outcome. The 90‐day follow‐up data (mRS) were collected by neurologists through either telephone or face‐to‐face interviews conducted with the patients or their family members.

### DWI measurement

DWI scans were most acquired using a PHILIPS Ingenia 3.0T MRI scanner, with the following acquisition parameters: a repetition time of 2506 ms, an echo time of 68 ms, a field of view measuring 230 × 230 mm, an image matrix of 152 × 102 pixels, b‐values of 0 and 1000 s/mm^2^, a section thickness of 5.0 mm, and an interslice gap of 1.0 mm. The DWI data were automatically uploaded to the Picture Archiving and Communication System (PACS), subsequently downloaded in DICOM format, and then submitted to the imaging laboratory for evaluation by two independent readers. Additionally, two neurologists manually delineated infarct regions in the DWI images using 3D Slicer software^17^ (version 4.11.2 and 5.2.2).

### Study outcomes

The primary outcome was functional independence, defined as achieving an mRS score range from 0 to 2 at the 90‐day follow‐up, in addition to evaluating mortality at the same time point. The safety endpoint is all types of intracranial hemorrhage, categorized according to the Heidelberg bleeding classification.^18^

### Statistical analysis

Demographic profiles, baseline clinical, and outcomes were compared between EVT and non‐EVT groups. Continuous variables were expressed as mean (standard deviation [SD]) for normally distributed parameters or median (interquartile range [IQR]) otherwise. While categorical variables were depicted through frequency and proportion. Univariate comparisons were executed through a chi‐squared test for categorical variables, and for continuous variables, unpaired *t*‐tests or Mann–Whitney *U*‐tests were employed, as appropriate.

The propensity score (PS) was calculated using a logistic regression model. The selection of weighting parameters for PS derivation was guided by clinical considerations and previous study,^19^ considering disease severity and comorbidity burden. These parameters included age, NIHSS score, infarct volume in DWI, and the occlusion site. PS matching was performed using a 1:1 pair matching without replacement employing the nearest‐neighbor matching algorithm with a caliper width of 0.2. After PSM, a comparative analysis was undertaken to evaluate differences in baseline characteristics and outcome variables. Categorical variables were examined with chi‐squared tests, while non‐normally distributed data were assessed using the Mann–Whitney *U*‐test. Continuous variables displaying a normal distribution were analyzed using the unpaired Student *t*‐test as the preferred statistical method.

PS adjustment was executed through the application of an inverse probability of treatment weighting (IPTW) strategy, aimed at approximating the impact of randomization and ameliorating the influence of confounding associated with the indication for endovascular treatment. Weights were allocated to the treatment modalities based on the inverse of the propensity score (1‐PS) for patients undergoing EVT and the inverse of the complement of the propensity score (1/[1‐PS]) for patients exclusively receiving medical management. All statistical analyses were conducted using IBM SPSS Version 26 software (Armonk, NY) and R version 4.2.1 software (Vienna, Austria). A significance level of *p* < 0.05 was established for statistical significance.

## Results

### Study population and baseline characteristics

**Figure 1:**
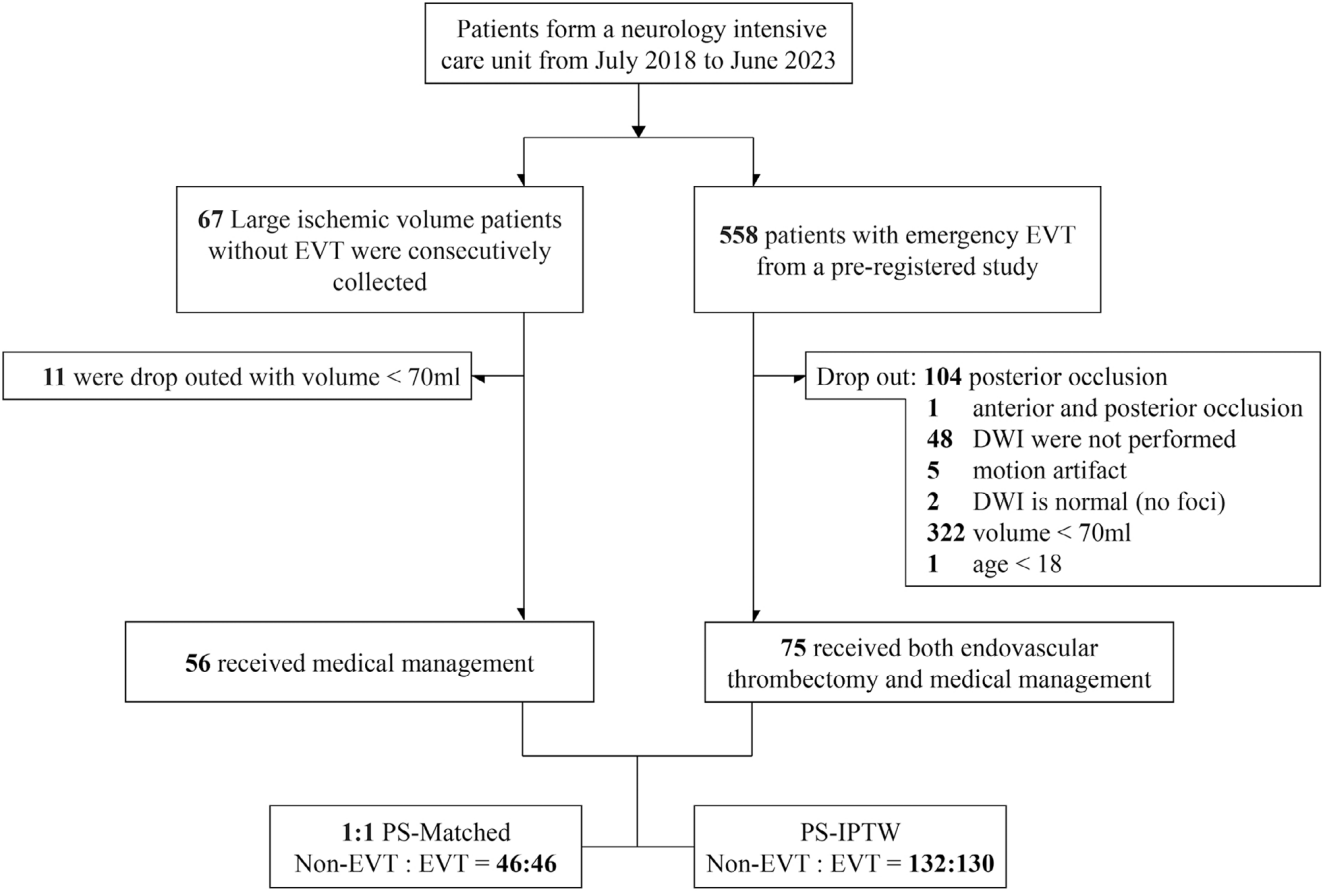
Study flow chart. DWI, diffusion‐weighted magnetic resonance imaging; EVT, endovascular thrombectomy; IPTW, inverse probability of treatment weighting; PS, propensity score.

From July 2018 to June 2023, a total of 558 EVT patients and 67 non‐EVT patients were enrolled in this study. Out of these, 75 EVT patients and 56 non‐EVT patients met the inclusion and exclusion criteria of the study (Fig. 1). Table 1 provides an overview of the demographic and baseline clinical characteristics of the study cohort based on the treatment received. The intraclass correlation coefficient for infarct volume between the two readers was 0.992 (*p* < 0.001). In the overall cohort, there were comparable median (IQR) NIHSS scores (EVT: 18 [14–21], non‐EVT: 17 [15–24]; *p* = 0.517), infarct volumes (EVT: 121.6 [95.7–164.9] mL, non‐EVT: 148.5 [101.5–224.2] mL; *p* = 0.099), proportions of female individuals (EVT: 44.0%, non‐EVT: 44.6%; *p* = 0.942), and medical history (previous stroke, hypertension, diabetes, atrial fibrillation), occlusion location, occlusion side, and wake‐up stroke across both treatment modalities. However, patients receiving EVT exhibited a lower mean (SD) age (EVT: 67.2 [14.2] years, non‐EVT: 73.5 [12.4] years; *p* = 0.008) and a shorter median (IQR) time from stroke onset to DWI (EVT: 305 [166–488] min, non‐EVT: 458 [268–709] min; *p* = 0.003). Furthermore, the proportion of current smokers was significantly elevated in the EVT group (EVT: 36.0%, non‐EVT: 14.3%; *p* = 0.005), and the administration of intravenous thrombolysis was more frequent in the EVT group (EVT: 33.3%, non‐EVT: 14.3%; *p* = 0.013). In the EVT group, 53.5% of patients experienced intracranial hemorrhage.

**Table 1:** Outcomes and baseline characteristics of study population.

	Total (*n* = 131)	Non‐EVT (*n* = 56)	EVT (*n* = 75)	*p* value	Test
Age, mean ± SD, y	69.9 ± 13.7	73.5 ± 12.4	67.2 ± 14.2	0.008*	Unpaired Student's *t*
Sex, *n* (%)					
Female	58 (44.3)	25 (44.6)	33 (44.0)	0.942	Pearson *χ* ^2^
Male	73 (55.7)	31 (55.4)	42 (56.0)		
History, *n* (%)					
Previous stroke	29 (22.1)	11 (19.6)	18 (24.0)	0.552	Pearson *χ* ^2^
Hypertension	77 (58.8)	36 (64.3)	41 (54.7)	0.269	Pearson *χ* ^2^
Diabetes	19 (14.5)	11 (19.6)	8 (10.7)	0.149	Pearson *χ* ^2^
Atrial fibrillation	70 (53.4)	32 (57.1)	38 (50.7)	0.462	Pearson *χ* ^2^
Hyperlipidemia	20 (15.3)	9 (16.1)	11 (14.7)	0.825	Pearson *χ* ^2^
Current smoker, *n* (%)	35 (26.7)	8 (14.3)	27 (36.0)	0.005*	Pearson *χ* ^2^
NIHSS score before EVT, median (IQR)	18 (15–22)	17 (15–24)	18 (14–21)	0.517	Mann–Whitney *U*
Occlusion location, *n* (%)					
ICA	66 (50.4)	27 (48.2)	39 (52.0)	0.668	Pearson *χ* ^2^
M1	50 (38.2)	23 (41.1)	27 (36.0)	0.554	Pearson *χ* ^2^
M2	15 (11.5)	6 (10.7)	9 (12.0)	0.819	Pearson *χ* ^2^
Occlusion side, *n* (%)					
Right	60 (45.8)	24 (42.9)	36 (48.0)	0.559	Pearson *χ* ^2^
Left	71 (54.2)	32 (57.1)	39 (52.0)		
Time from onset to DWI, median (IQR), min	358 (190–535)	458 (268–709)	305 (166–488)	0.003*	Mann–Whitney *U*
Infarction volume, median (IQR), mL	123.6 (97.0–196.6)	148.5 (101.5–224.2)	121.6 (95.7–164.9)	0.099	Mann–Whitney *U*
Wake‐up stroke, *n* (%)	34 (26.0)	18 (32.1)	16 (21.3)	0.163	Pearson *χ* ^2^
Intravenous rt‐PA or TNK, *n* (%)	33 (25.2)	8 (14.3)	25 (33.3)	0.013*	Pearson *χ* ^2^
Time from DWI to DSA first image, median (IQR), min	NA	NA	73 (57–97)	NA	NA
Cause of stroke, *n* (%)					
Embolic	NA	NA	63 (84.0)	NA	NA
Atherosclerotic	NA	NA	7 (9.3)	NA	NA
Tandem	NA	NA	2 (2.7)	NA	NA
Others	NA	NA	3 (4.0)	NA	NA
Procedural modes, *n* (%)					
ADAPT only	NA	NA	37 (49.3)	NA	NA
Stent retriever	NA	NA	28 (37.3)	NA	NA
Balloon or/and stenting	NA	NA	10 (13.3)	NA	NA
Reperfusion, *n* (%)	NA	NA	68 (90.7)	NA	NA
Hemorrhage, *n* (%)	NA	NA	40 (53.3)	NA	NA
mRS 0–2, *n* (%)	20 (15.3)	5 (8.9)	15 (20.0)	0.081	Pearson *χ* ^2^
mRS 0–3, *n* (%)	33 (25.2)	11 (19.6)	22 (29.3)	0.206	Pearson *χ* ^2^
mRS 6, *n* (%)	62 (47.3)	30 (53.6)	32 (42.7)	0.216	Pearson *χ* ^2^

ADAPT, a direction aspiration first‐pass technology; DSA, digital subtraction angiography; DWI, diffusion weighted imaging; EVT, endovascular thrombectomy; ICA, internal carotid artery; IQR, interquartile range; mRS, modified Rankin Scale; M1, M2, segments of the middle cerebral artery; NA, not applicable; NIHSS, National Institutes of Health Stroke Scale; rt‐PA, recombinant tissue plasminogen activator; SD, standard deviation; TNK, recombinant human TNK tissue‐type plasminogen activator.

* indicates statistical significance under the two‐sided *p* < 0.05 assumption.

### Functional and safety outcomes

In this cohort, 15 patients in the EVT group and 5 patients in the non‐EVT group achieved a favorable functional outcome (mRS 0–2) at 90 days. There was no statistically significant association between EVT and functional outcome at 90 days (EVT: 20.0%, non‐EVT: 8.9%, *p* = 0.081). Similarly, 32 patients in the EVT group and 30 patients in the non‐EVT group were dead at 90 days follow‐up, and EVT did not exhibit a significant impact on mortality (EVT: 42.7%, non‐EVT: 53.6%, *p* = 0.216).

### Functional and safety outcomes based on PS‐matched

**Figure 2:**
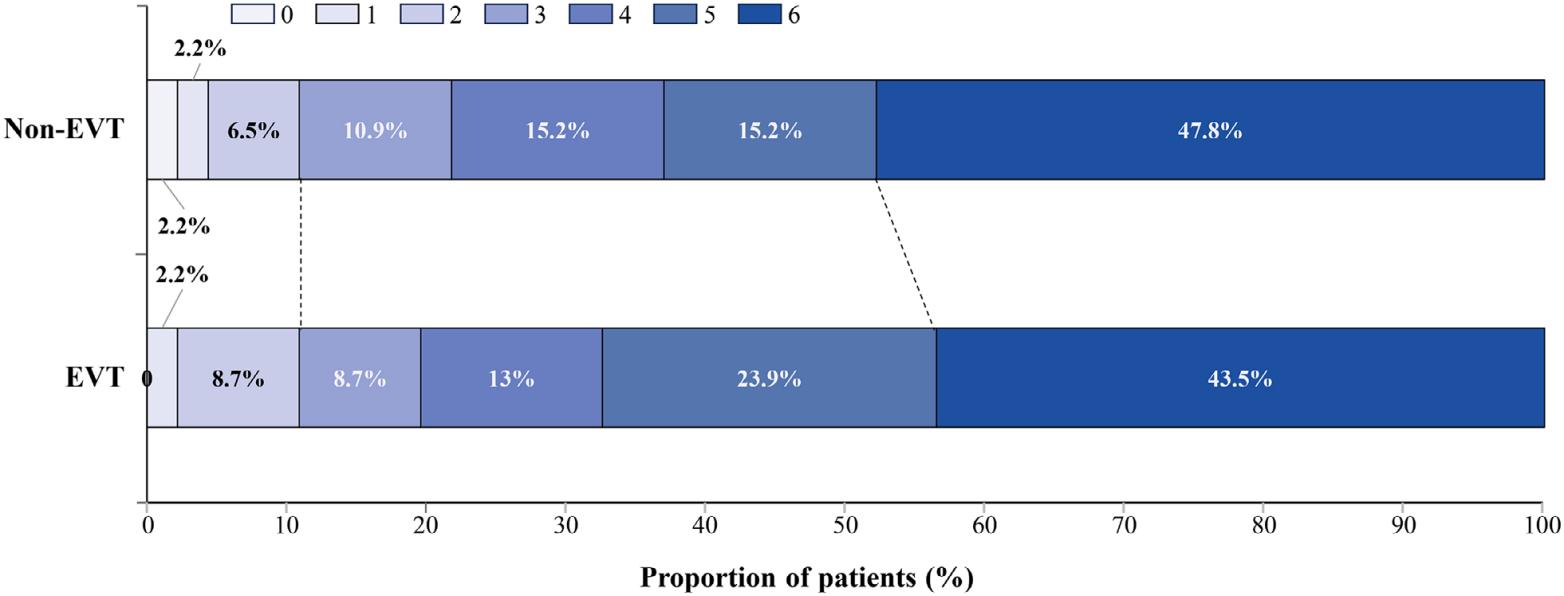
Distribution of modified Rankin Scale scores in each group at 90 days follow‐up after propensity score matching. 0 indicates no neurological deficit, 1 indicates no clinically significant disability, 2 indicates slight disability, 3 indicates moderate disability requiring some help, 4 indicates moderately severe disability, 5 indicates severe disability, and 6 indicates death.

**Figure 3:**
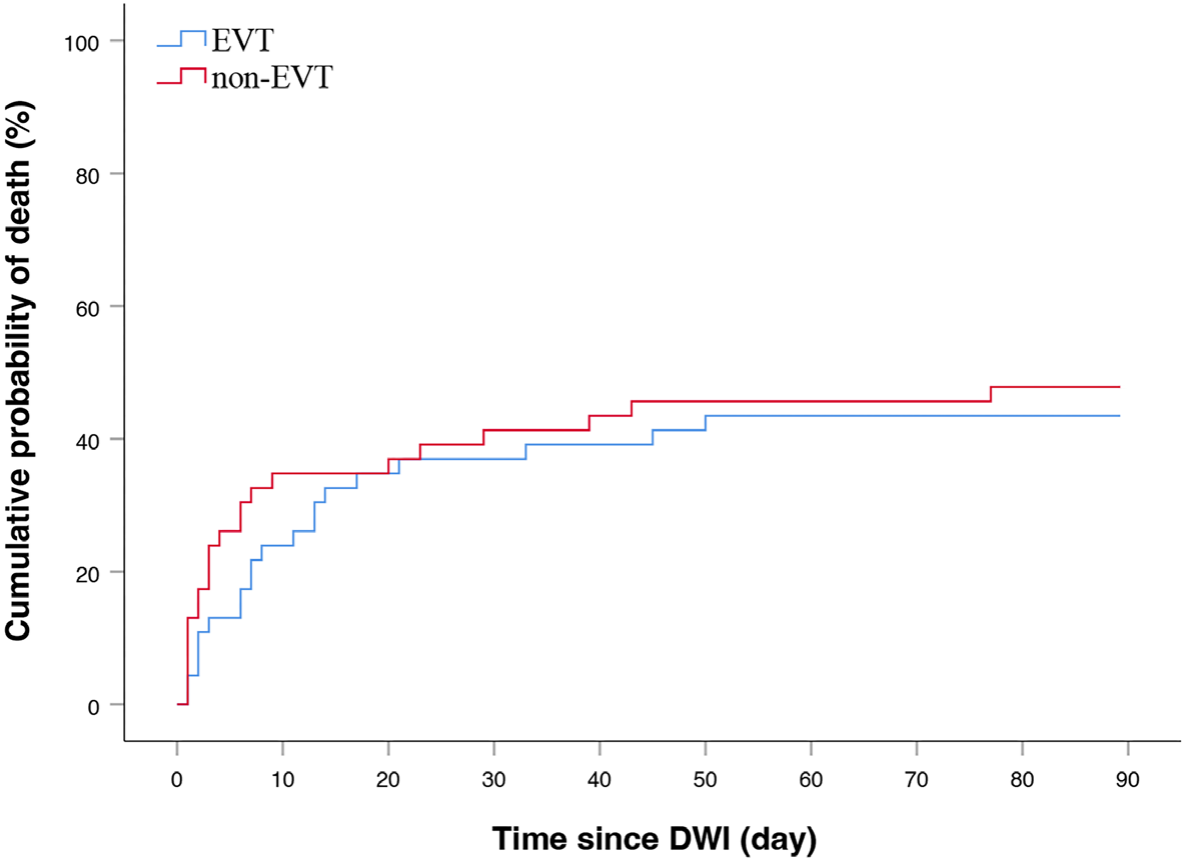
Kaplan–Meier plot of the cumulative risk of death in each treatment group after propensity score matching. DWI, diffusion‐weighted magnetic resonance imaging; EVT, endovascular thrombectomy.

When matching based on age, stroke severity, infarct volume and stroke location (Fig. S1), a total of 46 matched pairs were identified (Table 2). Patients who underwent EVT did not exhibit increased functional independence (EVT: 10.9%, non‐EVT: 10.9%; *p* = 1.000), and there was likewise no statistically significant disparity in mortality rates between the two patient groups (EVT: 43.5%, non‐EVT: 47.8%; *p* = 0.675) (Fig. 2). The Kaplan–Meier plot of the cumulative risk of death in each treatment group after PSM (Fig. 3) did not show a significant difference (*p* = 0.565).

**Table 2:** The overview of the outcomes for mRS 0–2 and mRS 6, which have been presented following PS‐matched and PS‐IPTW adjustments with age, NIHSS score, occlusion location, and infarction volume as covariates.

	PS‐Matched			PS‐IPTW		
	Non‐EVT (*n* = 46)	EVT (*n* = 46)	*p* value	Non‐EVT (*n* = 132^a^)	EVT (*n* = 130^a^)	*p* value
Age, mean ± SD, y	71.7 ± 12.4	71.4 ± 12.2	0.899	69.8 (13.5)	69.6 (13.6)	0.966
NIHSS score, mean ± SD	18.9 ± 8.2	18.3 ± 6.4	0.702	18.4 (7.7)	18.4 (6.1)	0.983
Occlusion location, *n* (%)						
ICA	23 (50.0)	22 (47.8)	1.000	60 (45.7)	62 (48.1)	0.796
M1	15 (32.6)	18 (39.1)	0.664	53 (40.1)	51 (39.3)	0.928
M2	8 (17.4)	6 (13.0)	0.772	16 (12.6)	19 (14.2)	0.812
Infarction volume, mean ± SD, mL	155.7 ± 81.6	151.9 ± 75.7	0.817	150.4 ± 78.5	151.7 ± 72.0	0.927
mRS 0–2, *n* (%)	5 (10.9)	5 (10.9)	1.000	18 (13.7)	21 (15.8)	0.767
mRS 6, *n* (%)	22 (47.8)	20 (43.5)	0.675	58 (44.0)	61 (46.8)	0.762

EVT, endovascular thrombectomy; ICA, internal carotid artery; IPTW, inverse probability of treatment weighting; M1, M2, segments of the middle cerebral artery; mRS, modified Rankin Scale; NIHSS, National Institutes of Health Stroke Scale; PS, propensity score; SD, standard deviation.

^a^ Patients who were weighted by IPTW based on the PS calculation were rounded to integers, including patients in the ICA\M1\M2 groups, and the proportion behind the numbers was still calculated using the original values.

### Functional and safety outcomes based on PS‐IPTW

Utilizing PS‐IPTW based on age, stroke severity, infarct volume, and stroke location (Fig. S2), a total of 262 patients (EVT: 132, non‐EVT: 130) were identified (Table 2). The group receiving EVT did not manifest superior outcomes in terms of achieving functional independence (EVT: 15.8%, non‐EVT: 13.7%; *p* = 0.767). Additionally, there was no observed reduction in mortality rates among EVT‐treated patients (46.8%) compared to those managed with medical care only (44.0%; *p* = 0.762).

## Discussion

In a real‐world, single‐center cohort of patients with acute ischemic stroke characterized by infarct volumes more than 70 mL resulting from anterior circulation large vessel occlusion, no statistically significant differences were observed in terms of functional independence and mortality compared to the non‐EVT group. Nonetheless, it is worth noting that there was an observable trend favoring the EVT group. Among patients of comparable age, stroke severity, infarct volume, and occlusion location, those undergoing EVT did not demonstrate superior functional independence or reduced mortality when compared to those receiving medical management alone.

In clinical practice, individuals with a LIV constitute a minority subgroup within acute ischemic stroke cases.^1^ Collecting evidence to address the optimal management of these patients presents a formidable challenge. Favorable outcomes derived from four recently published randomized controlled trials on LIC,^9^, ^10^, ^11^, ^12^ in addition to two ongoing, unpublished randomized controlled studies (LASTE trail [NCT03811769], TESLA trail [NCT03805308]), employed the CT‐ASPECTS as an inclusion criterion. In the clinical setting, it is noticeable infarct core volumes are highly variable within the same ASPECTS score, and this consequently leads to varying outcomes.^14^ Mehdi Bouslama noted that patients with low ASPECTS but limited baseline infarct volume achieve independence in nearly 40% of cases.^15^ In other words, this specific subgroup falls into the category of individuals characterized by a small infarct core and large hypoperfusion. Previous study^6^ have demonstrated that this population clearly benefits from EVT.

The concepts of LIC and LIV are distinct and not synonymous. Randomized controlled trials focusing on LIC, published after 2022, demonstrate the effectiveness of EVT.^9^, ^10^, ^11^, ^12^ The disparity between the findings of the current study and those of prior research can be attributed to variations in the enrollment criteria. In the RESCUE‐Japan study, a comparison of median infarct volumes between the EVT group and the non‐EVT group revealed values of 94 mL (IQR: 66–152) versus 110 mL (range: 74–140). In the SELECT2 study, the median infarct volumes were 82 mL (IQR: 56–89) for the EVT group and 86 mL (IQR: 84–104) for the non‐EVT group. In the ANGEL‐ASPECT study, these median volumes were 60.5 mL (IQR: 29–86) versus 63 mL (IQR: 31–86) for the EVT and non‐EVT groups, respectively. Functional outcomes (defined as an mRS score of 0–2) were observed among patients in the EVT group at rates of 14.0% in RESCUE‐Japan, 20.3% in SELECT2, and 30.0% in ANGEL‐ASPECT, indicating a strong correlation with infarct volume. In our investigation, the infarct volume, as determined by DWI, was 121.6 mL (95.7–164.9) versus 148.5 mL (101.5–224.2) initially. After PSM, it was shown to be 151.9 ± 75.7 mL for the EVT group and 155.7 ± 81.6 mL for the non‐EVT group, resulting in a 10.9% achievement of functional independence.

CT‐ASPECT scores exhibited limited concordance among neurologists,^14^ whereas DWI‐ASPECT scores demonstrated superior agreement.^20^ Moreover, the relationship between ASPECTS scores and infarct volume was not parallel, resulting in significant variations in infarct volumes among cases with the same ASPECT score. This was also observed in our previous study.^13^ The established golden standard for evaluating acute infarct volume is DWI,^21^, ^22^ notwithstanding occasional reports of post‐EVT high‐signal reversal in DWI, a phenomenon that is not prevalent. Like our approach, the RESCUE‐Japan study extensively employed MRI for diagnosing acute ischemic stroke. However, in the study's inclusion criteria, the DWI‐ASPECT score was utilized rather than infarct volume.

Contemporary assessments of infarct volume rely on identifying regions with cerebral blood flow (CBF) below 30%^9^, ^10^ in computed tomography perfusion (CTP). Nevertheless, utilizing CTP as a core infarction selection tool necessitates the utilization of artificial intelligence software for precise calculations. Moreover, the determination of infarct volume through CTP remains controversial due to its tendency to both overestimate and underestimate true core infarctions during the early stages.^23^ DWI, as an integral component of multimodal image screening, robustly delineates early core infarction and is regarded as the golden standard. Our observations revealed that regions displaying DWI lesions before EVT rarely exhibited subsequent reversal after EVT. Significantly, patients with high preoperative DWI signal intensity exhibited an increased susceptibility to the development of malignant brain edema.

Before statistical adjustments, patients in the EVT group showed a trend towards more favorable outcomes (EVT: 20.0%, non‐EVT: 8.9%, *p* = 0.081). However, it is noteworthy that patients in the EVT group were younger (EVT: 67.2 ± 14.2 years, non‐EVT: 73.5 ± 12.4 years, *p* = 0.008) and had a smaller infarct volume (EVT: 121.6 mL [95.7–164.9], non‐EVT: 148.5 mL [101.5–224.2], *p* = 0.099) before statistical adjustments. Given the nature of this real‐world study, neurologists routinely exercised their clinical judgment to select candidates with a more favorable prognosis. For instance, the younger age of patients in the EVT group might introduce a bias towards better outcomes. Following the baseline data matching process, this inclination towards EVT associated with improved functional outcomes disappeared. Considering the higher prevalence of smoking among patients in the EVT group and its association with atherosclerosis, it is plausible that it may contribute to increased procedural complexity. Our concern is that the indiscriminate expansion of EVT inclusion for LIC may lead to harmful consequences. Due to the inherent subjectivity associated with CT‐ASPECT scoring, direct neuroimaging indicators, such as low density, exhibit reasonable consistency. However, indirect indicators, such as the loss of insular ribbon sign and loss of gray‐white interface, may yield varying interpretations among different neurologists. As a result, this difference may result in biased evaluations, with neurologists possibly showing a preference for EVT in patients.

The use of ASPECT score as an inclusion criterion for thrombectomy in patients with LIC has yielded positive outcomes. However, the overall prognosis rate remains low and the majority of patients undergoing thrombectomy experience poor prognoses, with up to one‐third of them dying during 90 days. Experienced neurologists have adopted individualized recommendations based on preoperative evaluation, rather than a uniform approach for all patients. For this reason, there is an urgent need to undertake an individualized preoperative evaluation for thrombectomy in LIC, in order to avoid unnecessary surgical intervention for patients who are unlikely to benefit from this treatment.

## Limitations

This study has several limitations. First, this study was conducted within a single‐center setting and follows a non‐randomized approach. Second, almost all patients with an infarct volume < 70 mL underwent EVT; therefore, there were few patients in the non‐EVT group within 24 h, resulting in an imbalance between the two groups before statistical processing. Third, the sample size for this study is relatively small, given the low incidence of patients with a DWI volume greater than 70 mL in the early stages after stroke onset. Fourth, manual delineation of the infarct region is a labor‐intensive process and can be challenging to integrate into an emergency workflow. The use of artificial intelligence to identify infarct areas may offer a promising solution to this dilemma. Fifth, perfusion images from MRI were not included in this study. Once the infarct volume exceeds 70 mL, the penumbra becomes relatively small, and the mismatch becomes less significant. The DWI examination results in a delay in the time from stroke onset to artery puncture, whereas the rapid sequence of the 3.0T MR scanner serves to reduce this delay. Sixth, the mean signal intensity ratio (SIR)^24^ in patients who underwent thrombectomy was 1.12 ± 0.13. However, we did not utilize the signal intensity ratio (SIR) as a quantitative analysis index for selecting participants, potentially impacting outcomes in large infarct volumes after thrombectomy. Finally, patients in the non‐EVT group had a low completion rate of 24‐h follow‐up CT, and statistics on intracranial hemorrhage in the non‐EVT group could not be performed. The incidence of any intracranial hemorrhage at 53.3% in the EVT group may be a significant factor affecting outcomes in the EVT group.

## Conclusion

In a real‐world, single‐center, population‐specific study caused by large vessel occlusion in the anterior circulation, EVT in patients with infarct volumes more than 70 mL does not yield patient benefits. A prospective randomized controlled trial is warranted.

## Author Contributions

NNH conceived and collected the data and wrote the manuscript. XBZ, YZ, and YL performed the data analysis. HJM, HMG, YFW, SLL, XDY, TFL, YLW, JM, and WZS involved in manuscript review and revision. GJZ, YT, and MZC critically revised the report and accepted full responsible for the overall content. All authors contributed to the article and approved the submitted version.

## Conflict of Interest Statement

The authors declare that they have no known competing financial interests or personal relationships that could have appeared to influence the work reported in this paper.

## Funding Information

Funding for this study was supported by Natural Science Basic Research Project of Shaanxi Province (2022JM‐452).

## Supporting information


Figure S1.

Figure S2.

## Data Availability

The data supporting the findings of this study can be obtained from the corresponding author upon a reasonable request.
