# Supplementary figures and images for: Endovascular thrombectomy versus medical management on outcomes with infarct volumes more than 70 mL
Source: Ann Clin Transl Neurol. 2024 Jun 10;11(8):2040–8. doi: 10.1002/acn3.52124 (PMC11330213; doi:10.1002/acn3.52124)

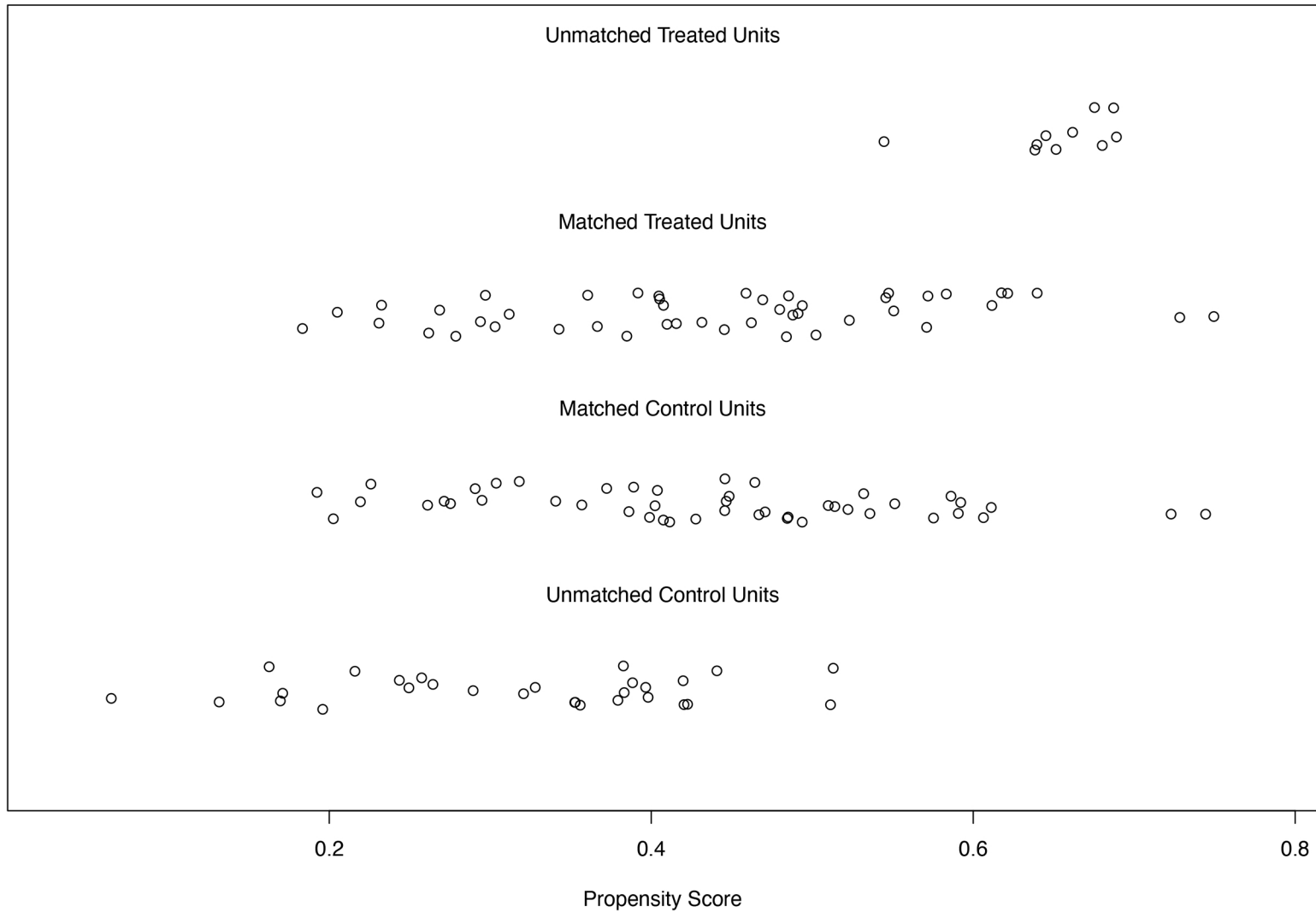

Supplement: Supplementary file 1 — Figure S1. [file ACN3-11-2040-s002.pdf]

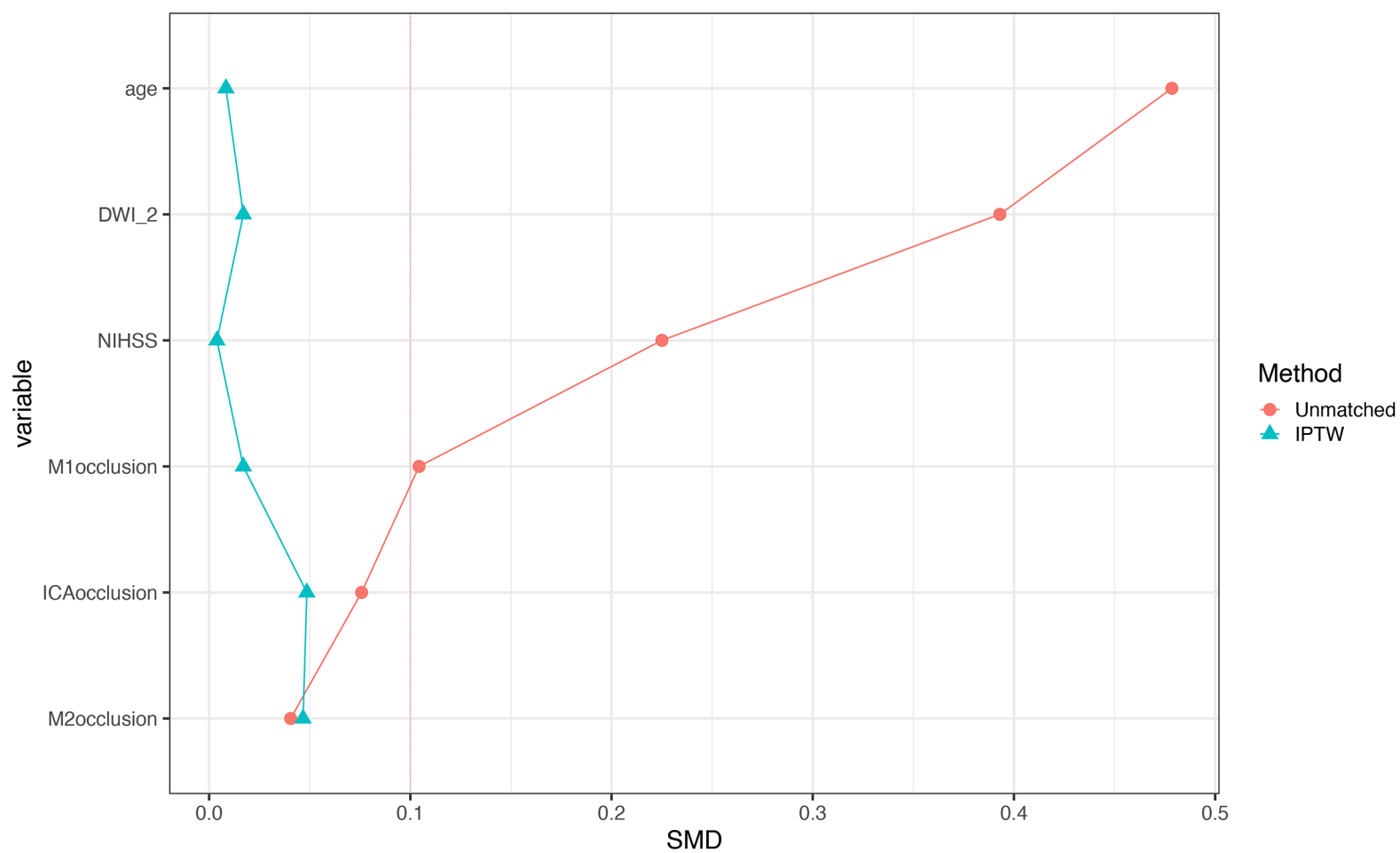

Supplement: Supplementary file 2 — Figure S2. [file ACN3-11-2040-s001.pdf]
